# Distribution of *Triatoma dimidiata* sensu lato (Reduviidae: Triatominae) and Risk Factors Associated with Household Invasion in Northern Belize, Central America

**DOI:** 10.1093/jme/tjab227

**Published:** 2022-01-22

**Authors:** Angela T Caranci, John P Grieco, Nicole L Achee, David F Hoel, Kim Bautista, Russell King, V Ann Stewart, Jittawadee Murphy, Penny Masuoka, Cara H Olsen

**Affiliations:** 1 Northwest MVCD, 1966 Compton Avenue, Corona, CA 92881, USA; 2 Department of Preventive Medicine & Biostatistics, Uniformed Services University of the Health Sciences, 4301 Jones Bridge Road, Bethesda, MD 20814, USA; 3 Eck Institute of Global Health, University of Notre Dame, 120 Brown Hall, Notre Dame, IN 46556, USA; 4 Ministry of Health, East Block Independence Plaza, Belmopan, Belize

**Keywords:** *Triatoma dimidiata*, Chagas disease, vector surveillance, sylvatic

## Abstract

To date, *Triatoma dimidiata* sensu lato [Reduviidae: Triatominae (Latreille 1811)] remains the sole vector species associated with Chagas disease transmission reported from Belize. Human infection data are limited for Belize and the disease transmission dynamics have not been thoroughly investigated, yet the likelihood of autochthonous transmission is supported by the widespread collection of infected vectors from within local households. Here, we report updated infection rates of the vector population and infestation rates for villages in north and central Belize. Overall, 275 households were enrolled in an ongoing vector surveillance program. Of the 41 insects collected, 25 were PCR positive for *T. cruzi*, indicating an infection rate as high as 60%. To further characterize the epidemiological risk of human–vector contact, determinants of household invasion were modeled. Local households were surveyed and characterized with respect to over 25 key factors that may be associated with household infestation by *T. dimidiata* s.l. While final models were not strongly predictive with respect to the risk factors that were surveyed, likely due to the low number of collection observations, the presence of domestic/peri-domestic dogs, nearby light sources, and household structure materials could be the focus of continued risk assessments. In northern Belize, this vector survey lends support to *T. dimidiata* s.l. inhabiting sylvatic settings as opposed to the classical paradigm of domiciliated vector populations. This designation has strong implications for the local level of human exposure risk which can help guide vector surveillance and control resources.

Chagas disease continues to be one of the most widespread, neglected tropical diseases in Latin America despite several regions reporting decreased incidence of human infections throughout endemic areas of Central and South America (Nouvellet et al. 2015). Although varying levels of success have been attained in controlling Chagas disease over the last 40 years, the biology of transmission and control of the insect vectors must remain a focus of current research to maintain this decline (Coura and Dias 2009, Peterson et al. 2019). *Trypanosoma cruzi* (Trypanosomatida: Trypanosomatidae (Chagas 1909)) is the causative agent of Chagas disease and is vectored by true bugs of the order Hemiptera in the family Reduviidae, subfamily Triatominae (Hotez et al. 2007). Following contact with feces of infectious invertebrate vectors, *T. cruzi* parasites can enter and infect human hosts resulting in a spectrum of disease symptoms referred to as Chagas disease (Andrade 1991). The spectrum of disease can range from asymptomatic infection to chronic disease affecting the tissues of major organs including the heart, esophagus, and rectum, resulting in an estimated annual loss of 430,000 Disability-Adjusted Life Years (DALYs) (Nouvellet et al. 2015).

Chagas disease has been listed among the major neglected tropical diseases largely due to a lack of adequate diagnostic tools and treatment protocols (Hotez et al. 2007). In Latin America, the disease is second to malaria in the amount of area that is endemic for the parasite, causing an estimated 8 million current infections and threatening approximately 109 million people who live at risk (WHO 2014). Because no vaccine is available, many nations have employed blood screening and case detection to prevent spread of the parasite by congenital transmission or blood transfusion (Dias 2015). The success gained in decreasing the incidence of Chagas disease in the New World has varied due to differences in the efficacy of surveillance and control methods focused on insect vectors across varied ecological settings throughout the large endemic region (Coura and Dias 2009). Control efforts have reported success where transmission is predominantly due to domesticated vector populations; however, widespread sylvatic populations require the need for control programs that are integrated and targeted to local transmission ecology (Dumonteil et al. 2009).

Belize, on the eastern coast of Central America (Fig. 1), is one of several nations with limited surveillance of Chagas disease transmission. In this region, information related to human cases of Chagas disease as well as the infection and transmission dynamics of the insect vectors is largely lacking. Lainson (1965) was the first to report *T. cruzi* in Belize, known as British Honduras at that time. Later, Petana and Coura (1967) reported finding *T. cruzi* in *T. dimidiata* s.l. collected from the central region of the country. The first human infection reported in the literature was from the same central area in Cayo District of Belize (Petana 1978). To date, the only cross-sectional serologic study focusing on human disease incidence in Belize compared immigrant populations to local, healthy military personnel via the analysis of blood donations (Jamarillo et al. 1997). The authors reported only one serologically positive sample from a Belizean citizen, stressing the prominence of imported cases from bordering regions of Central America that were simultaneously recorded from non-native patients in Belize. Animal reservoir populations from Belize have been found infected with *T. cruzi* since 1969, at which time *T. dimidiata* s.l. was also implicated as the most likely vector (Petana 1968). The authors provided the only comprehensive study focusing on the possible animal reservoir species implicating coati, opossums, and rodents as likely reservoirs (Petana and Coura 1967). A single recent study regarding *T. cruzi* transmission in Belize, limited to the southern and central districts, used community-based collections to report an infection rate of 28% in the *T. dimidiata* s.l. population (Polonio et al. 2009). Together these studies reveal the presence of both pathogen and vector, as well as, evidence of domestic human infection, necessitating additional research to better define local vector ecology. Since the planning and implementation of this data collection occurred, additional clarification of the diversity of species within the *T. dimidiata* s.l. complex has been published; however, additional molecular analysis of samples collected during this project is pending (Justi et al. 2018). It should also be noted that a separate species has been described from within country which was formerly referred to as *T. dimidiata* s.l., but samples from the described species location were not included in the pathogen infection analysis herein (Dorn et al. 2018).

**Fig. 1.**
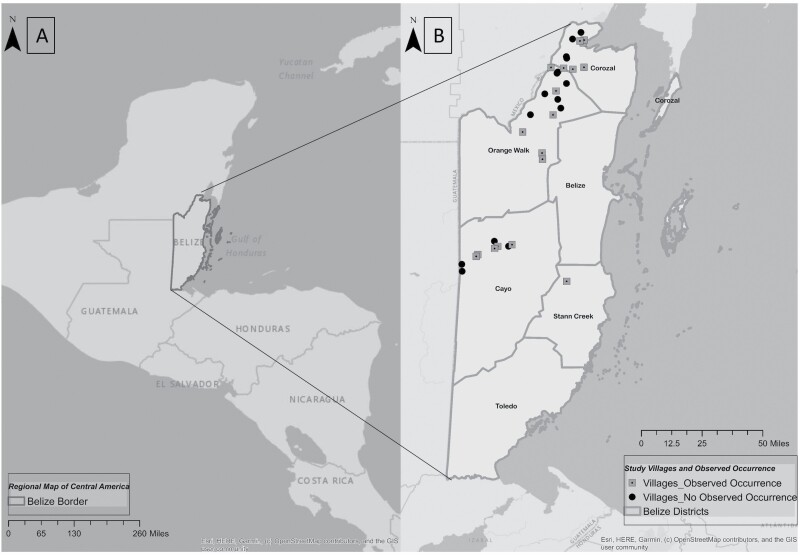
**Belize and administrative districts.** (A) The left image displays the location of Belize in the context of Central America. (B) The administrative districts of Belize, this study investigated households in the northern districts of Corozal and Orange Walk, and centrally, Cayo. Circles represent villages included in the surveillance portion of this study that did not report vector observations during this study, while squares signify included villages with observed presence of *T. dimidiata* s.l. in the household.

The general objective of this study was to assess and report on Chagas disease vectors in regions of northern Belize where such information was largely unknown. Multiple methods of capturing the distribution of local triatomines were assessed. Collected specimens positively identified as vectors were analyzed for *T. cruzi* infection. Household risk factors were modeled to determine association with the presence of invading *T. dimidiata* s.l. The information gained should inform local health and vector control offices on the risks associated with Chagas disease vectors as well as provide officials with preliminary methods for strengthening surveillance and control programs.

## Materials and Methods

### Study Area and Collection Period

Specimen collection occurred within the two most northern administrative districts of Belize, Corozal District, and Orange Walk District (Fig. 1), from November 2012 to September 2014. The more centrally located Cayo District (Fig. 1) was added in June of 2013 with collection continuing through September 2014. After enrollment into the study, all households were revisited for sampling within the same four-week period and repeated at three-month intervals thereafter. Any household that was not able to be sampled at any two recurring visits was discontinued as loss to follow up. Initially, 210 houses were randomly selected within 23 villages throughout Corozal and Orange Walk Districts in order to evaluate collection methodologies. Each village was mapped, and quadrants were added to scale with the size of the area, houses were approached for study inclusion in each quadrant. Villages were included based on input from local Vector Control District officials with the aim of surveilling a wide extent of the target region. Inclusion of the Cayo District added an additional 65 households across 8 villages to the total number of collection sites (total 275 enrolled). Collection method, described in more detail below, were assigned to each household based on the willingness of residents, as some collection methods were much more invasive than others. Regardless of the collection method performed at a given location, an adult member of each household was presented with an educational pamphlet regarding the transmission of Chagas disease and the associated disease vectors reported from surrounding regions (Supp Material 3 [online only]: color pictures provided for reference). After informed consent was granted through an ongoing vector collection program conducted through the Belizean Ministry of Health, staff stressed the importance of avoiding direct contact when handling any possible vectors. Our use of data was approved through the Belizean Ministry of Health IRB process.

### Vector Collection Methods

**Fig. 2.**
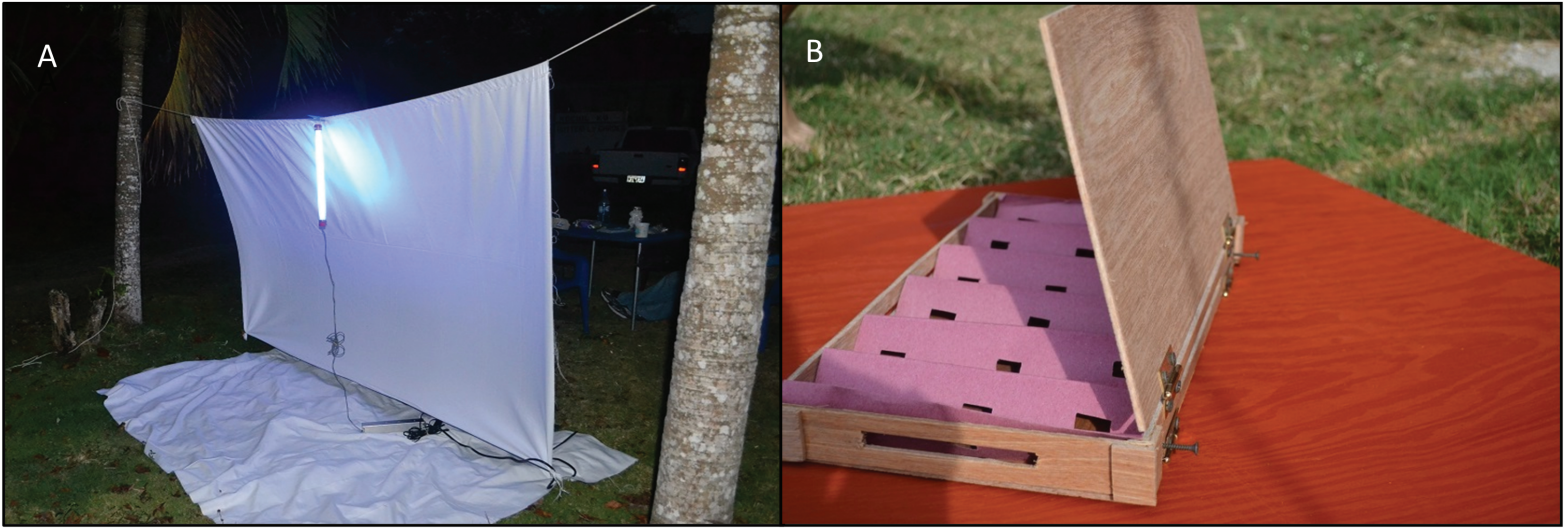
**Example collection methods.** Two of the four types of collection methods pictured for reference. (A) Nocturnal lighting setup used in season 1 and discontinued thereafter. (B) Sensor boxes constructed in country from common wood panels used as housing construction material used in season 1 and discontinued thereafter.

Commonly, the cool dry season in Belize stretches from November to early February, with the strong heat and rains occurring from May through September (National Meteorological Service of Belize 2012). At the onset, four common collection methods were adapted from the literature and employed at various locations in each village (Fig. 2). The first method, referred to hereafter as ‘community collection’ involved community-assisted surveillance (Dumonteil et al. 2009, Polonio et al. 2009). Each household was provided collecting materials and again coached on the importance of minimizing contact when collecting any insects that resemble the vectors of interest. Cooperating household members were asked to collect any insects that resembled the reference images provided in the educational pamphlet and to record the date and location of collection on the specimen container. New pamphlets and collection materials were offered at each return visit throughout the study period. The second collection method employed across all villages was referred to as active searching. Active searching has been a common means of locating and collecting vectors associated with household domiciliation (Coura and Dias 2009). In the interest of standardizing this collection method to account for the different size and types of households present within and across local communities, the time allotted for searching each house included 20 min per common use household area and an additional 5 min per bedroom. During this time, 2 trained entomologists, employed and trained by the Belizean Ministry of Health, simultaneously searched the indoor area. Additionally, another trained entomologist concurrently searched the peridomestic area for an allotted 25-minute period. This process was performed during each visit for one year. The third collection method involved the placement of sensor boxes (Dumonteil et al. 2002). This means of surveillance is meant to offer shelter to domiciliated vectors and allow for the passive detection of vectors within the household. Plywood boxes (14 × 25 × 9 cm) lined with accordion-folded construction paper were placed at each location at one of three possible locations: behind headboard of bed, behind other large furniture, or in roof rafters (this was often guided by discretion of household inhabitants to ensure limited tampering) and inspected upon subsequent visits for one year. The final collection method involved nocturnal lighting as described in Rebollar-Tellez et al. (2009). Ultra-violet (15 W) tube lights (BioQuip Products Inc., Rancho Dominguez, CA) were hung against white canvass sheets (3 × 3 m) and monitored every 30 min for the 12 h period from dusk until dawn. Due to lack of vectors collected by means of active searching, sensor box surveillance, and nocturnal lighting, these methods were discontinued as primary collection methods after the first year of the study. Community collection was then employed at all sites for the duration of the study. Specimens there were collected live via active searching and nocturnal lighting were immediately stored in 80% isopropyl alcohol. Community collected specimens were stored dry until retrieval and transferred to alcohol when received during visits and molecular analyses were performed 6–8 months after collection from field. All specimens were identified using a morphological key (Lent and Wygodzinsky 1979) and confirmed by PCR of the ITS-2 region as previously described by Richards et al. (2013).

### Determination of T. cruzi Infection Status of Vector Specimens

Because of the lag period occurring between collection periods, most specimens were too desiccated to attempt determination of infection status by microscopic examination of fecal matter. Therefore, for each specimen, the terminal segment of the abdomen was removed using sterile dissection tools. Individual samples were then pulverized in PBS using a hand operated, cordless pellet pestle with sterilized tissue grinders (Fisher Scientific, Pittsburgh, PA). The slurry was then processed by PCR to determine *T. cruzi* presence according to methods reported in Virreira et al. (2003). DNA was extracted from these samples using QiaAmp DNA Blood Mini Kit (QIAGEN, Valencia, CA). Separate PCR reactions were run to amplify sequences targeting both genomic DNA (TCZ) and kDNA minicircle segments (S35/36). PCR products were run on 1.5% agarose gels stained with 0.5 µg/ml ethidium bromide. Samples from which both target sequences were successfully amplified were considered positive for *T. cruzi*.

### Collection of Household Attribute Data

At the time of enrollment and after informed consent was obtained, adult inhabitants were given a brief survey that inquired on the individual's previous encounters with *T. dimidiata* s.l. as well as additional behavioral information regarding household use of insecticides. Additional information regarding the visual household structure and presence of domesticated animals was recorded. The attributes recorded included: GIS location, number of rooms, number of bedrooms, presence of screened doors, presence of screened windows, presence and type(s) of animals in domestic area, presence and type(s) of animals in peridomestic area, external light sources, community light sources, distance to community light sources, status of surrounding vegetation (primary, secondary, agricultural), wall material, roof material, floor material, number of wall gaps larger than 2 cm, and opening between roof eaves and wall structure. Later, the distance from each house location to the village periphery was measured using Google Earth (Google Earth Pro 2012).

### Data Analysis

Presence data were mapped at the village level using ArcGIS 10.1 software (ESRI, Redlands, CA). Village infestation rates were calculated as the percentage of household positive for vector presence. District infestation rates were calculated as the percentage of villages positive for vector presence(Dumonteil et al. 2002). Chi square analysis tested trends in vector presence for sex differentiation and seasonality. Univariate analysis of each attribute was performed to determine correlation of each household characteristic monitored (Supp Material 2 [online only]) with observations of *T. dimidiata* s.l. within the household (SPSS 28, Armonk, CA). Characteristics with a significance value less than 0.2 were included in the final multiple logistic regression. Stepwise regression was also employed using the variables in Table 2 in an attempt to standardize variable selection in a final model.

## Results

### *T. dimidiata* s.l. Distribution

A total of 41 *T. dimidiata* s.l. specimens were collected across three administrative districts throughout the collection period. On occasion, vector specimens were submitted to local district-level Belize Ministry of Health: Vector Control offices by residents. Although these submissions were not part of our initial collection protocol, they were subsequently tracked to the associated village, georeferenced, and included in the distribution maps reported in order to be as inclusive as possible but were not included in infestation or dispersion characteristics or household risk factor analysis as the attribute data were not available. All of the specimens collected were adults; no eggs or nymphs were reported throughout this collection period. Approximately 54% of the adults collected were male with the remaining 46% female. Of the total specimens collected, the distribution among collection methods was as follows: community collection *n* = 38; active searching *n* = 2; sensor box surveillance *n* = 0; and nocturnal lighting *n* = 1. Village and district infestation rates are reported in Table 1, and range from 10 to 40% and 14 to 64% respectively. The geographic locations of the villages that were samples, and those resulting in collection of *T. dimidiata* s.l. can be seen in Fig. 2. Throughout the collection period, 13 specimens were collected from February to May 2013–2014, 17 specimens from June to September 2012–2014, and 11 specimens from October to January 2012–2014. Collection timing was designed with the aim of capturing seasonal trends; however, there was no significant trend in the seasonal distribution of reported vector presence (Chi-square *P* > 0.05).

**Table 1.** Villages reporting presence, number of *T. dimidiata* s.l. (n) and village-level infestation index

	Village level distribution of *T. dimidiata* in Belize								
	Corozal District			Orange Walk District			Cayo District		
	Name	n	Infestation index	Name	n	Infestation index	Name	n	Infestation index
Village	Ranchito^*b*^	4	30%	Indian Church^*a*^	8	40%	Santa Elena	3	20%
	Buena Vista	2	20%	San Carlos	5	20%	Blackman Eddy	2	10%
	Xaibe	2	10%	Douglas	2	20%	Esperanza	2	13%
	San Andres	2	10%	Trial Farm	1	10%	Unitedville	2	NA
	Caledonia^*b*^	1	10%	Guinea Grass	1	10%	Camelote	1	13%
	Progresso	1	10%	San Felipe	1	10%	Middlesex^*c*^	1	NA
District	Avg. Village Infest	15%		Avg. Village Infest	18%		Avg. Village Infest	14%	
	District Infestation	60%		District Infestation	46%		District Infestation	50%	
	*T. cruzi* Infection Rate	58%		*T. cruzi* Infection Rate	61%		*T. cruzi* Infection Rate	64%	

All other observations were from community assisted collections. District level data including average village infestation index, district infestation rate, and *T. cruzi* infection rate.

^*a*^Denotes the village that recorded a single nocturnal lighting collection.

^*b*^Denotes villages with successful active search collections.

^*c*^The single collection was turned into Cayo District MoH but later traced back to Stann Creek District.

### *T. cruzi* Infection Rates of Vector Populations

The overall infection rate of the target vector population was 61%. Infection rates by district ranged from approximately 58% to 64% and are reported in Table 1. Due to the low number of individual vectors collected, we were not able to report any trends associated with district level distribution or seasonal presence and *T. cruzi* positive *T. dimidiata s.l.*

### Risk Factors Associated with Household Invasion

Of the 41 specimens collected during the study, 38 were associated with households enrolled into the survey portion. Due to the fleeting nature of the vector's presence in households, it is important to report on trends associated with household characteristics that are local to northern and central Belize. The survey data reported that 80% (*n* = 203) of households lacked screened doors, with 66% (*n* = 166) of houses having no or only partially screened windows. Only 6% (*n* = 15) of households reported housing an animal within the home overnight, while 83% (*n* = 209) of households reported ownership of animals kept in the surrounding peridomestic area. The most common animal associated with the peridomestic setting was dogs (78%, *n* = 196) and chickens were the second most common (37%, *n* = 93). All but one village was equipped with community lighting in the form of streetlights that were kept on throughout the night. Household structure materials are also commonly associated with vector presence. In this study region, the most common wall material was cement (46%, *n* = 118) followed by treated wood (41%, *n* = 103). Roof material was most commonly made of sheets of corrugated zinc (83%, *n* = 209). Locally acquired thatch and vegetation material were rarely used as the main household structure material (5%, *n* = 13). Additionally of note, interview data showed that 87% (*n* = 219) of household adults surveyed had no prior knowledge or experience with *T. dimidiata* s.l. as pictured in the educational pamphlet. The use of some form of commercially available insecticide occurred in 88% (*n* = 221) of surveyed households.

Of the total number of houses initially enrolled (*n* = 275), 23 (8%) were lost to follow up and only 26 household locations were positive for *T. dimidiata* s.l. presence. Univariate analysis explored the relationship between each household risk factor surveyed and the presence of *T. dimidiata* s.l. reported from within the household. Of the attributes, nine were included in multivariate analysis (Table 2) using the *P* < 0.2 threshold. These variables were included in the final regression; however, the resulting model was not statistically significant. Therefore, the multivariate factors included were unable to reject the null hypothesis that the risk factors listed in Table 2 taken together have no effect on household vector invasion. Similarly, stepwise regression was not able to successfully build a model with significance (*P* < 0.343).

**Table 2.** Household risk factors

		Vector absent	Vector present	Odds ratio	Multiple regression sign.
Screen door	Absent	194	15	2.847	0.049
	Present	42	8		
Domestic animal	Absent	221	22	0.676	0.703
	Present	15	1		
Dog presence	Absent	61	1	8.546	0.021
	Present	175	22		
Community lights	Absent	80	9	0.798	0.523
	Present	156	14		
CommLight distance	5 m	6	0	1.038	0.992
	10 m	41	6		
	20 m	72	6		
	30 m	38	2		
	Absent	79	9		
Wall type	Cement	108	10	1.07	0.865
	Zinc	3	3		
	Thatch	27	0		
	Wood	98	10		
Roof type	Cement	25	2	0.516	0.123
	Zinc	197	18		
	Thatch	12	0		
	Wood	2	0		
Floor type	Cement	198	19	1.195	0.673
	Zinc	2	0		
	Thatch	9	0		
	Wood	27	4		

Household risk factors included in multivariate regression based on univariate regression significance where *P* < 0.20. Multivariate analysis could not successfully build a model that statistically predicted household invasion of *T. dimidiata* s.l. Contingency tables and odds ratios for factors of interest below.

## Discussion

The design of a successful vector control program principally depends on investigations into the distribution and ecology of the target vector species. This initial reporting sought to not only provide some basic data to support a growing vector control program, but also formally document the infestation rates and infection status of triatomine vectors of Chagas disease in Belize. Widespread surveillance of villages in the northern and central region of Belize resulted in the collection of *T. dimidiata* s.l., which remains the sole vector reported from this Central American nation. Despite the seemingly scant local vector population reported here, the infection rate in specimens collected within households was 60%. This rate is higher than previously documented (Polonio et al. 2009) but supported by literature reporting vector infection rates greater than 50% (Schofield 2000). Similar infection rates were also reported when modeling the relationship between vector abundance and population infection rate in the bordering Yucatan region of Mexico (Dumonteil and Gourbiére 2004). Authors reported a regionally specific inverse relationship between the density of local vector populations and *T. cruzi* infection rates. It is important to note that currently the reported incidence of human–vector contact is low, as local *T. dimidiata* s.l. populations are highly sylvatic. However, with the high infection rates of the local populations, fluctuations in the sylvatic setting that impact vector behavior may lead to increased human contact with infectious vectors. This study is limited by a lack of host feeding data and future bloodmeal analysis of specimens encountered in the domestic and peridomestic settings could continue to inform the risk of endemic human infection. Sylvatic *T. dimidiata* s.l. populations have been reported from elsewhere in Central America, where adult vectors invade the household area from surrounding secondary brush vegetation and agricultural plots (Monroy et al. 2003). This complex relationship requires additional and ongoing surveillance to fully determine any risk factors associated with vegetative patterns in Belize and elucidate possible shifts that may impact human risk.

At this early point in our understanding of potential *T. cruzi* transmission in this region, a few key observations should be noted. Throughout two collection seasons, only 41 specimens were collected despite the use of four different surveillance methodologies in an attempt to capture this vector species. Community collection efforts were by far the most efficient means of collecting *T. dimidiata* s.l. in the region, using this study design. The village and district level infestation rates were low compared to neighboring regions in the Yucatan peninsula, where village infestation rates averaged 47% and district infestation averaged 83% (Dumonteil et al. 2002). All of the 41 specimens collected were adults; therefore, there is little evidence of household domiciliation of this vector population in the northern districts and it is likely that the presence of vectors within human habitations is transient or intrusive as defined by Waleckz et al. (2015). Because the local vector population does not seem to display behavior associated with regular household infestation, it may also be possible that Belizeans living within this region are not as familiar with the vector itself, although printed educational materials and pinned specimens were offered to familiarize residents with vector characteristics, additional outreach materials would only serve to strengthen community-based collections in future endeavors. Of the people surveyed in this study, 87% reported never having previously seen *T. dimidiata* s.l. prior to enrollment in this study. Not only was this a likely reflection of the small size of local vector populations, but also a low level of local familiarity with this neglected disease and associated vectors. Continued surveillance and educational interventions at the village level are needed to ensure full capture of *T. dimidiata* s.l. distribution throughout this region, particularly when relying on community collection (Dumonteil et al. 2009, Polonio et al. 2009). Similarly, ecological trends associated with seasonality of household invasion may emerge as the reporting of local vector populations improves.

The fleeting, non-domiciliated nature of the vector population in northern Belize seems similar to that reported in neighboring areas of Yucatan, Mexico where climate and ecology are comparable (Dumonteil et al. 2013). Many of the household risk factors investigated in this study were chosen based on previously reported associations with *T. dimidiata* infestation (Polonio et al. 2009, Dumonteil et al. 2013). As reported, the northern region of Belize does not currently sustain fully domiciliated populations of *T. dimidiata* s.l. This may be due to the nature of household structures that are common throughout the region. Currently, most homes are composed of cement, treated wood, and zinc materials. The thatch roofing and locally sourced logs that have reportedly been associated with *T. dimidiata* s.l. infestation were present in less than 5% of households included in this study (Petana 1978). Of the household characteristics recorded, eight were significantly associated with household infestation based on univariate analysis (Supp Material 2 [online only]). As reported in other studies, the results presented here support the association between the presence of dogs in the domestic or peridomestic setting, as well as the relative location of community lighting, and the collection of *T. dimidiata* s.l. within the household (Dumonteil et al. 2013). The relationship between peridomestic animals leading to increased risk for vector invasion relies on the premise that these transient, bloodmeal seeking vectors may be attracted to additional hosts. Another factor potentially associated with *T. dimidiata* s.l. presence was the household distance to community light sources. Pacheco-Tucuch et al. (2012) published controlled chamber tests supporting the hypothesis that *T. dimidiata* s.l. are attracted to white light during nocturnal hours, as well as field data showing that houses closer to streetlights were more likely to be infested. The trend associating community light sources with the presence of *T. dimidiata* s.l. was further supported by Dumonteil et al. (2013). It is important to note that in this study, the presence of a community based light source was recorded if that source was mounted within 30 m of an enrolled household. This may have implications for village-level control interventions targeting *T. dimidiata* s.l. if additional research can determine possible preferential behavior associated with specific wavelengths of light. However, any trends based on this univariate analysis should only be considered in the context of 26 households reporting presence of *T. dimidiata* s.l. Multivariate analysis was unable to build a statistically significant model based on the variables of concern including those discussed above: dog presence and proximity to streetlights. Additional work tracking the vector to human exposures in this region must be performed in order to better understand not only the disease risk to the local human populations, but also the comparative vector ecology of potential subspecies in the greater Central American context (Dorn et al. 2018).

In conclusion, we report the widespread distribution of *T. dimidiata* s.l. throughout northern and central Belize. Despite low levels of household invasion, the infection rates of local vector populations averaged 60%. Therefore, despite the likelihood of human–vector contact being low, the risk for human infection remains. Factors such as the presence of dogs in the peridomestic setting and community light sources within 30 m of the house are potential predictors of household invasion by *T. dimidiata* s.l. This study was an initial investigation into the ecology of *T. dimidiata* s.l. in this setting and should be used to strengthen vector surveillance programs. However, it is important to note that the collection work was limited in the cumulative number of observations and the majority of collections relied on community participation which imparts sampling bias and potentially recency bias. Some household attributes were not monitored, including domestic income, number of fulltime residents, and elevation of households above ground level. Additional human case data and bloodmeal analysis are crucial in calculating human risk. We suggest that more research be performed in order to determine the additional ecological factors of land use and animal reservoir populations on the risk of Chagas disease in Belize.

## Supplementary Material

tjab227_suppl_Supplementary_Material_1

tjab227_suppl_Supplementary_Material_2

tjab227_suppl_Supplementary_Material_3
