# Supplementary figures and images for: Distribution of Triatoma dimidiata sensu lato (Reduviidae: Triatominae) and Risk Factors Associated with Household Invasion in Northern Belize, Central America
Source: J Med Entomol. 2022 Jan 22;59(2):764–71. doi: 10.1093/jme/tjab227 (PMC8924975; doi:10.1093/jme/tjab227)

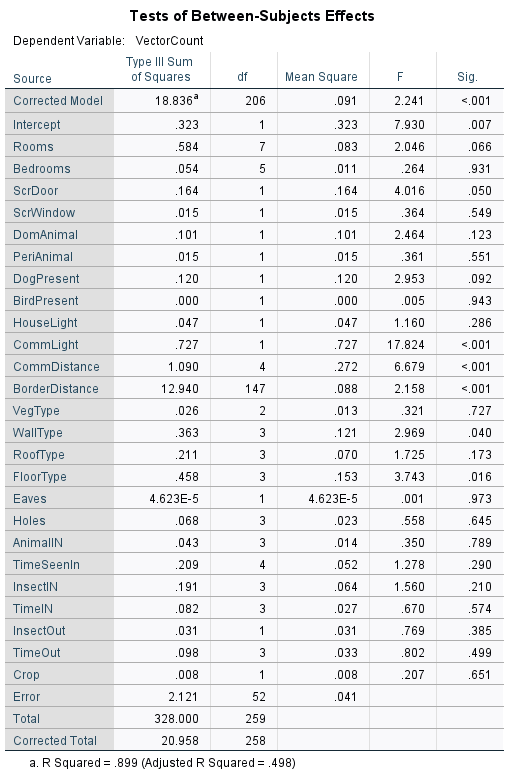

Supplement: tjab227_suppl_Supplementary_Material_2 [file tjab227_suppl_supplementary_material_2.docx]
