# Supplementary material for: Distribution of Triatoma dimidiata sensu lato (Reduviidae: Triatominae) and Risk Factors Associated with Household Invasion in Northern Belize, Central America
Source: J Med Entomol. 2022 Jan 22;59(2):764–71. doi: 10.1093/jme/tjab227 (PMC8924975; doi:10.1093/jme/tjab227)

How can you prevent Chagas disease?

Better living conditions

- Change thatch material roofing to zinc, wood, concrete etc.
- Repair all cracks on the walls of your house
- Do not leave firewood, lumber, bricks or other construction material lying around the house
- Replace dirt floor with cement or wood flooring

House cleanliness

- Always maintain your house clean
- Avoid sleeping with animals inside the house
- Keep beds away from walls

Finding the bug

- Check firewood and other materials for the "Bug"
- Check in and around your house frequently for the "Bug"

BUT AVOID DIRECT HANDLING OF THEM!

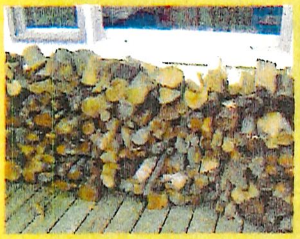

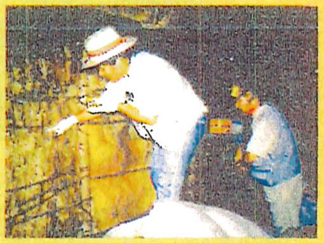

Avoiding the disease

Find and take all "Bugs" to the nearest Health Facility

1). When capturing bugs, always protect your hands with a plastic bag. Use the same bag to store the bug.

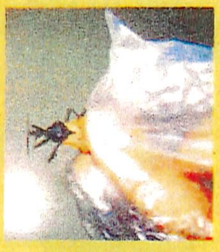

2). On a piece of paper, write down the name and address of the head of the family and date of capture.

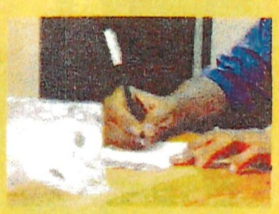

3). Put the piece of paper inside the plastic bag

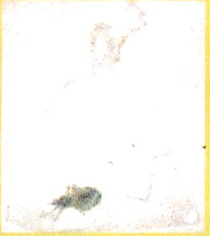

4). Properly wash hands.

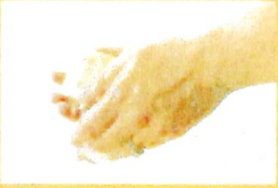

5). Take the plastic bag to the nearest Health Facility

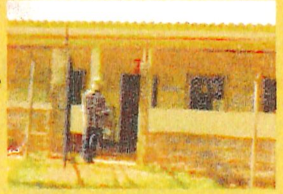

National Vector Control Program
Ministry of Health Belize

Chagas Disease

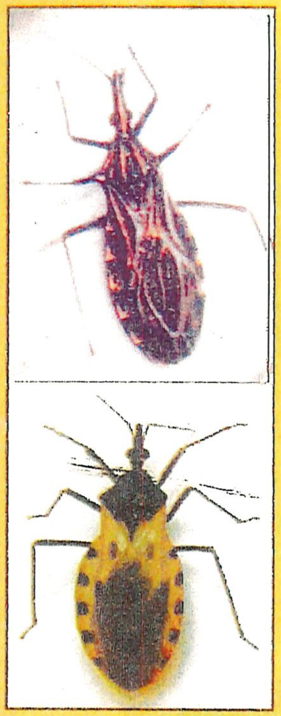

What you need to know

Chagas is a disease which is transmitted by the ***Triatoma dimidiata***, which feeds on blood. This bug is also known as the **"Kissing Bug"**

MINISTRY of HEALTH
equal health for all

Pan American Health Organization
Regional Office of the World Health Organization

What is Chagas disease?

It is a disease transmitted by a chinch that feeds on blood.

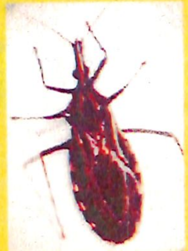

Rhodnius Prolixus

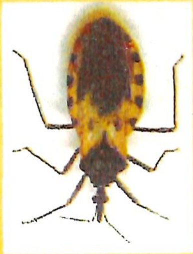

Triatoma Dimidiata

What are the signs/symptoms of the disease?

One to 2 weeks after becoming infected the following signs/symptoms appear:

- Fever and shivering
- Tiredness
- Most cases are asymptomatic

After the parasite enters the body, the eye may become swollen for 4-6 weeks. This swelling is known as Romaña's sign.

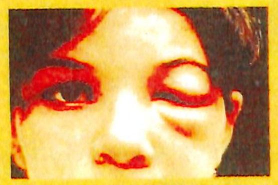

A lesion on the skin may form where the parasite penetrated the skin, this is known as a chagoma.

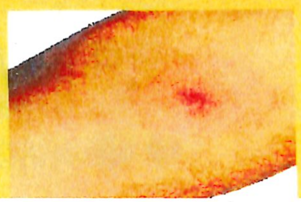

After 10-30 years heart damage becomes evident which could lead to death.

Where can the bug be found?

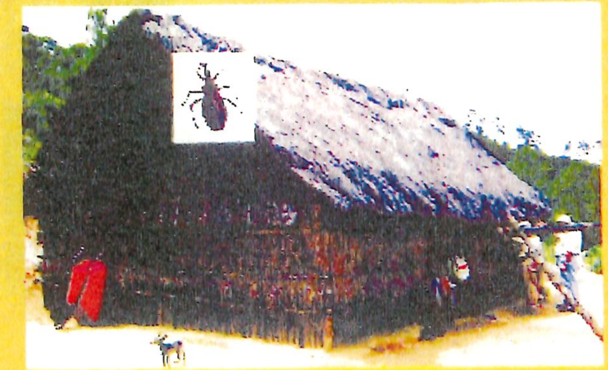

Rhodnius Prolixus

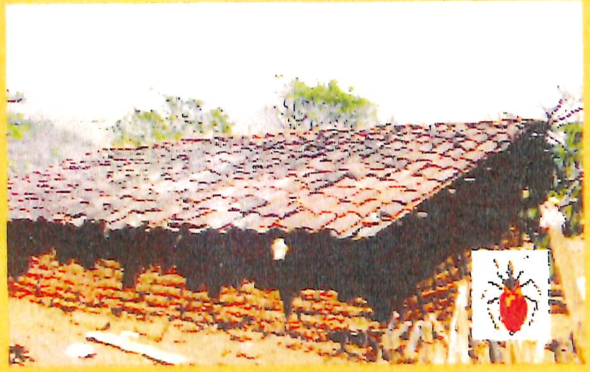

Triatoma Dimidiata

Preferred sites for the bug

1). Thatch or other natural roofing.

2). Behind or underneath furniture

3). Behind wall paintings and fixtures.

4). Under wooden homes and firewood piles

5). Between cracks in the walls of buildings

6). Door and window frames.

7). Chicken coop, pig pens, stables, barns etc.

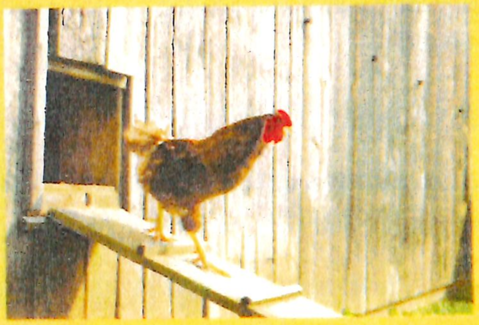

Chicken coops are a common place to find the bug.

How is the disease transmitted?

1). The parasite is located in the faeces of the bug. It defecates on the spot where it has bitten. When scratching the spot the parasite enters the body through the wound.

NOT ALL BUGS ARE INFECTED

2). Infected blood transfusion.

3). It can also be transmitted from an infected pregnant woman to her unborn child.

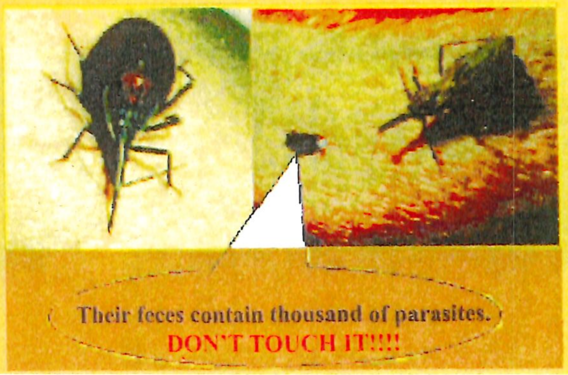

Supplement: tjab227_suppl_Supplementary_Material_3 [file tjab227_suppl_supplementary_material_3.pdf]
